# Supplementary figures and images for: Interaction between the assembly of the ribosomal subunits: Disruption of 40S ribosomal assembly causes accumulation of extra-ribosomal 60S ribosomal protein uL18/L5
Source: PLoS One. 2020 Jan 27;15(1):e0222479. doi: 10.1371/journal.pone.0222479 (PMC6984702; doi:10.1371/journal.pone.0222479)

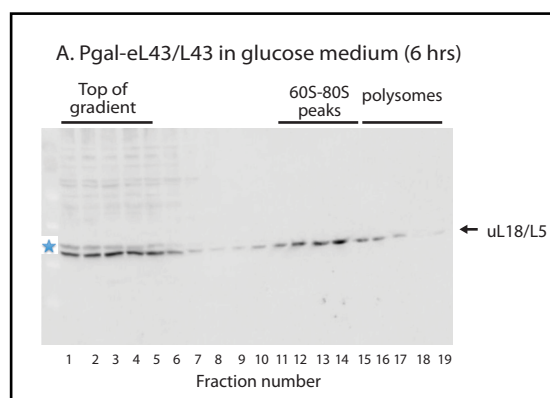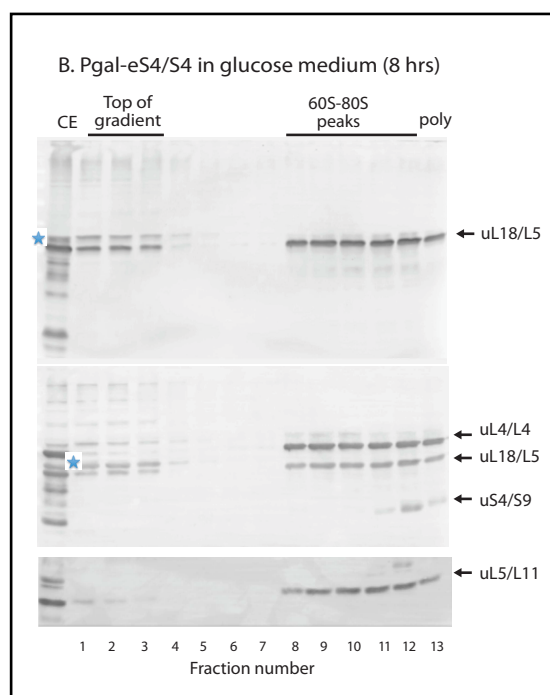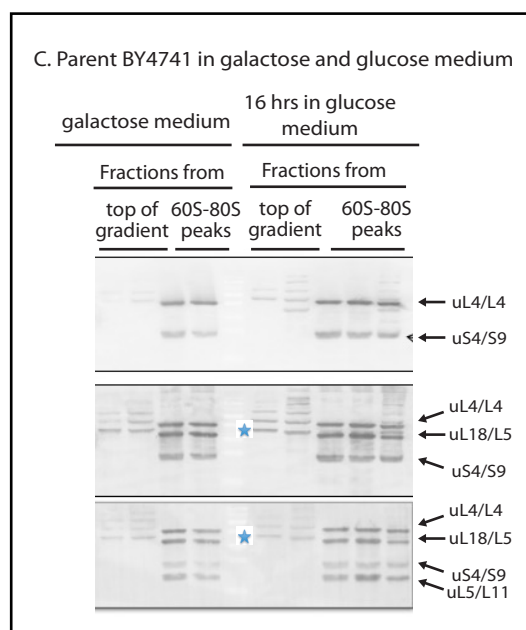

Uncropped images of westerns for Fig 2

Supplement: S1 Fig — (PDF) [file pone.0222479.s001.pdf]

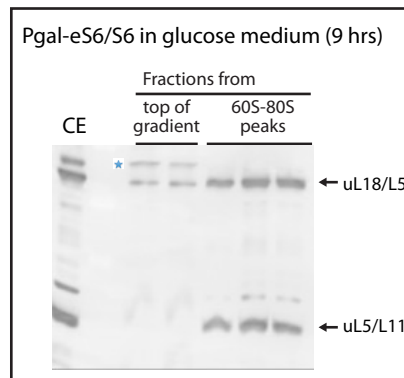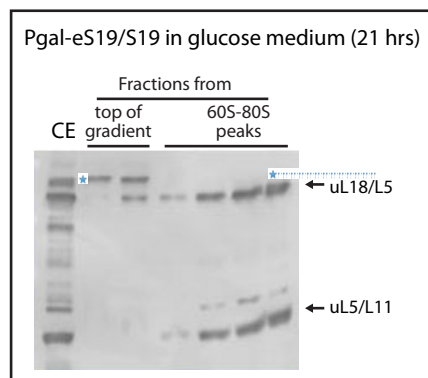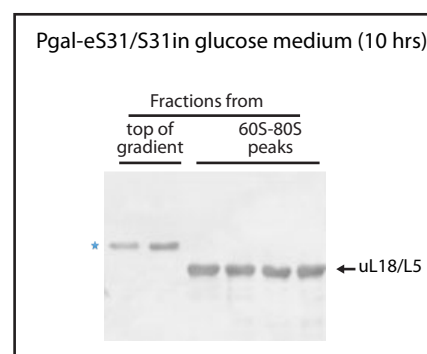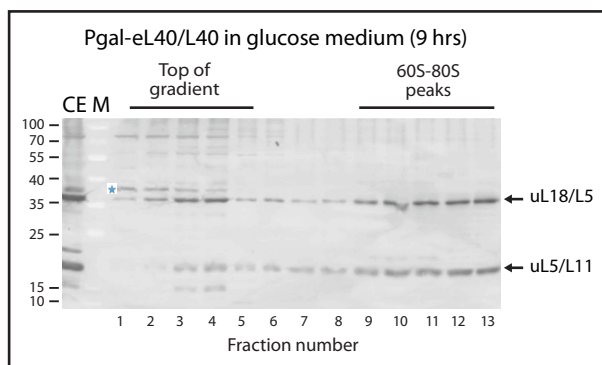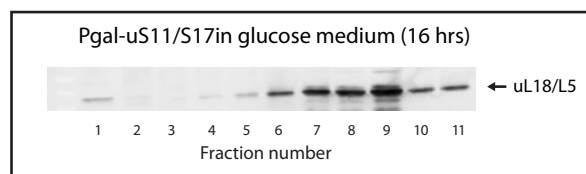

For Pgal-eS4, see Fig 2B  
Pgal-eL43/L43 in  
glucose medium (6 hrs),  
see Fig S1

Uncropped images for Fig 3A

Supplement: S2 Fig — CE whole cell extract. M molecular weight markers in kD. (PDF) [file pone.0222479.s002.pdf]

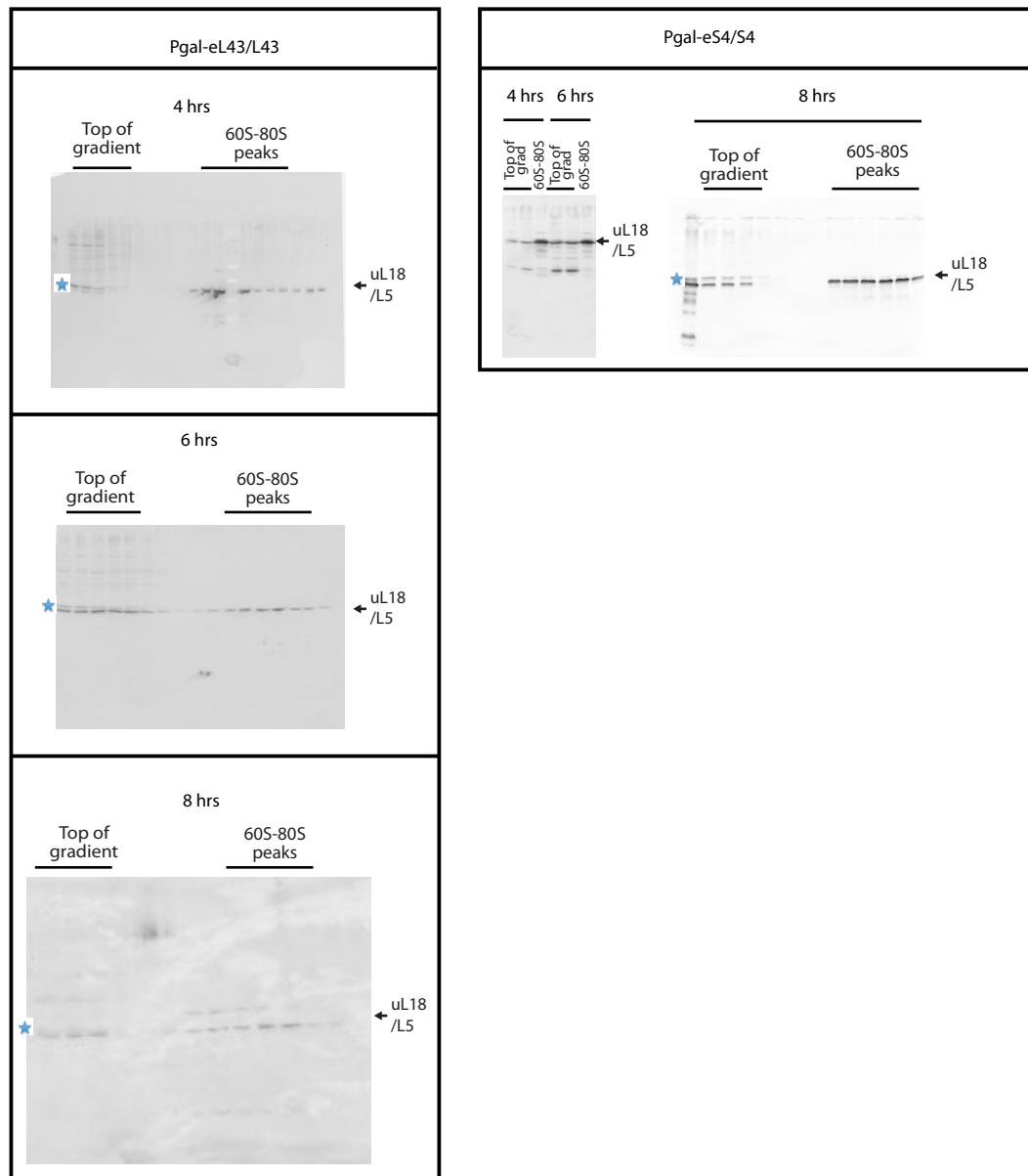

Uncropped Westerns for Fig 4

Supplement: S3 Fig — (PDF) [file pone.0222479.s003.pdf]
